# A longitudinal study of the association between depression, anxiety and stress symptoms of university students in Serbia with excessive social media use before and during COVID-19 pandemic

**DOI:** 10.3389/fpubh.2023.1140961

**Published:** 2023-12-18

**Authors:** Aleksandar Višnjić, Kıvanç Kök, Jovana Višnjić, Tamara Jovanović, Roberta Marković

**Affiliations:** ^1^Faculty of Medicine, University of Niš, Niš, Serbia; ^2^Institute of Public Health of Niš, Niš, Serbia; ^3^Department of Biostatistics and Medical Informatics, International School of Medicine, Istanbul Medipol University, Istanbul, Türkiye; ^4^Research Institute for Health Sciences and Technologies (SABITA), Istanbul Medipol University, Istanbul, Türkiye

**Keywords:** social media, BSMAS, DASS42, COVID-19 pandemic, depression, anxiety, stress, mental health

## Abstract

**Background:**

Besides the well known good side of social media, it cannot be denied some of its negative effects. This two-phase study aimed to find out whether the usage of social media during the COVID-19 pandemic showed some significant association with depression and anxiety symptoms, and levels of stress.

**Methods:**

The study was based on the survey of 1,476 randomly selected students at the initial phase (December 2019 to February 2020), and 1,400 students of the same cohort at the follow up phase (December 2021–February 2022). The collected data included socio-demographics, social media usage aspects, and the ones concerning levels of depression, stress and anxiety symptoms. Standardized questionnaires – the Bergen Social Media Addiction Scale (BSMAS) was applied to measure the levels of social media addiction, and the Depression Anxiety Stress Scale (DASS 42) was administered to evaluate the symptoms of depression, anxiety and stress.

**Results:**

The comparison of responses regarding the six components of online social media addiction, which constitute the BSMAS, between the two phases of the study showed significant difference (*p* < 0.01) in favor of the follow up phase in the raised scores of all but one component. The probable severe or extremely severe symptoms of depression, anxiety, and stress were notably enhanced during the peak of pandemic, and all three of them were positively correlated with all 6 BSMAS components (*p* < 0.01). The results also indicate that students, who consumed alcohol beverages and psychoactive substances more frequently in the pandemic, exhibited more noticeable symptoms of depression (*p* < 0.01, for both associations). Higher levels of anxiety symptoms in students were found to be associated with longer sleep during the night (*p* < 0.01), increased alcoholic beverages consumption (*p* < 0.01). Stress levels had the strongest correlations with consuming alcoholic beverages during the pandemic (*r* = 0.16, *p* < 0.01).

**Conclusion:**

The comparison between two phases of this follow-up study revealed significant changes in the Internet usage characteristics, which may have had an essential influence on the investigated symptoms of depression, anxiety, and stress. However, other factors that may have influenced student’s mental health during the COVID-19 pandemic should also be considered.

## 1 Introduction

The basic assumption of our research is that the state of mental health of all people has worsened during the pandemic. Concurrently, people have been exposed to excessive daily news and media overload, extensively covering the course of the pandemic (with numbers such as performed PCR tests, incidence and mortality due to COVID-19). In addition to this, many public health professionals have been in touch with the mainstream media to inform about how to live with restrictions during this period.

The World Health Organization member states implemented the common recommended and additional epidemiological measures, with the aim of preventing the transmission of the virus and containing the contagion. However, little attention was paid to the negative effects of these measures on the mental health of the population, especially of the younger ones. There is a growing body of literature and reports demonstrating increase in the levels of depression and anxiety symptoms in most people during this time (1–11).

A very interesting study from Iran confirms that the most important COVID-19-related factors associated with depression, and anxiety are “having a vulnerable person in the family, risk of disease, and following COVID-19 news” (12). Ridley et al. (13) went a step further and claimed that people from the poorer strata of society were more exposed to the manifestation of mental disorders during the pandemic. Relatedly, sleep disturbances and insomnia among students were often reported in the literature, which aligns well with the mentioned health issues (14, 15).

However, unlike others, Marić et al. claim (nationally-representative, face-to-face survey in Serbia) that there were not any evidence that the prevalence of mental disorders exceeded the range of pre-pandemic data reported in the existing literature. They stated that “Covid-related stressors, although frequently reported, did not dramatically influence the prevalence of mental disorders” (16).

Remarkably, social media became a part of people’s everyday life long before COVID-19 pandemic. It used to be mostly a tool for social interaction, and entertainment. Additionally, it has emerged as an opportunity to develop an online business. However, the social media use is accompanied by potential negative consequences on psychological health, including its addictive potential (17). Since adolescence is a period of increased vulnerability for low self-esteem and the onset of depression and anxiety, it is essential to understand how social media use relates to these factors (18, 19).

This relationship between social media and mental health has become increasingly complex during the COVID-19 pandemic. Social media usage has been shown to increase in situations of natural disaster and other crises. Therefore, the COVID-19 pandemic may have created severe threats to global mental health (2). Social distancing measures, enforced on people because of COVID-19, led to loneliness that was associated with future mental health problems. The strongest association was with depression (2). This may be particularly due to the importance of the peer group for identity and support (that people could find on social media during pandemic) during this developmental stage (20).

A comparative meta-analysis study has found that for children and adolescents, their post COVID-19 probable anxiety and depression levels were significantly higher than the pre-pandemic period (21). Because of the social restriction that was emphasized during the pandemic, children and adolescents could not leave their homes for a long time. It has also been reported that anxiety and depression can be transmitted between household members, especially from parents to children (22). The risk of domestic violence in isolation period may also increase (23).

The aim of this study was to find out if the excessive social media use during the COVID-19 pandemic was increased, and whether it was correlated with worse depression and anxiety symptoms, as well as the elevated levels of stress. This work implemented a two-phase longitudinal study design and focused on university students.

## 2 Methods

### 2.1 Participants

This two-phase longitudinal study was performed at the University of Niš (Serbia) from December 2019 to February 2020 (the period of the first phase), and again from December 2021 to February 2022 (the period to the second phase). Standardized surveys were applied to evaluate the participants. The study included 1,476 randomly selected students from the faculties of biomedical sciences, law, economy, and various engineering and humanities sciences at the initial phase (namely Phase 1) (762 females–51.6% and 714 males–48.4%), and 1,400 students of the same cohort (692 females and 708 males) at the follow up (namely, Phase 2) (December 2021–February 2022).

In total 24,047 students were studying at the University of Niš during the first phase of this study. In order to obtain a confidence level of 95%, the minimum sample size (number of students) was computed as 379. The survey was conducted in the university amphitheaters.

### 2.2 Procedure

Levels of symptoms of depression, anxiety, and stress were evaluated by applying the Depression Anxiety Stress Scale (DASS 42) (24, 25). Symptoms of excessive social media use were measured by the Bergen Social Media Addiction Scale (BSMAS) (26, 27). Levels of depression symptoms were classified as following: normal (0–9), mild (10–13), moderate (14–20), severe (21–27), extremely severe (28+). Levels of anxiety symptoms were categorized as following: normal (0–7), mild (8–9), moderate (10–14), severe (15–19), extremely severe (20+). Levels of stress were: normal (0–14), mild (15–18), moderate (19–25), severe (26–33), extremely severe (34+).

The students also reported their socioeconomic characteristics, as well as answered the questions related to some of their lifestyle habits. Trained assistants applied the survey in the university amphitheaters. The duration was planned to be maximum of 30 min, including the time required for instructions. Only completely answered questionnaires were returned to the authors.

### 2.3 Measures

The Bergen Social Media Addiction Scale (BSMAS) was used for measuring social media addiction (26, 27). The BSMAS consists of six items assessing the core patterns of behavioral addictions, quantified by a five-point Likert scale (1 = very rarely or never, 2 = rarely, 3 = sometimes, 4 = often, 5 = very often), and also a five-point Likert from 1-strongly disagree to 5-strongly agree, concerning experiences associated with social media usage during the past year (26, 27). These 6 items elucidate the six core elements of addiction: Salience, Tolerance, Mood Modification, Relapse, Withdrawal Symptoms, and Conflict. (1) Salience – the activity that become the most important in one’s life and dominates one’s thinking, feelings, and behavior; (2) Tolerance – growing amounts of the activity are needed to achieve previous effects; (3) Mood Modification – the activity modifies mood; (4) Relapse – a tendency to revert to previous patterns of the activity after abstinence or control; (5) Withdrawal – the emergence of unpleasant feelings when the activity is discontinued or suddenly reduced; and (6) Conflict – the activity causes conflicts in relationships, in work or education, and other activities. The reliability of BSMAS based on the Cronbach’s alpha value was found as 0.84.

The DASS42 is a collection of 3 self-report scales focusing on depressive symptoms, anxiety, and stress. The subscales were designed to assess negative emotional states. Each of those three scales comprises 14 questions (24, 25). The participating students from the University of Niš were asked to rate each item so that obtained points reflect the extent to which they experienced each of the listed conditions during the week before the survey, from 0 (never) to 3 (mostly or almost always). The score results of depression and anxiety were calculated by adding the points for each related scale. The result was then computed for every respondent and for each of the subscales, based the score matrix, and then assessed as per the severity-rating index. The reliability scores, in the form of the Cronbach’s alpha values, rate the depression, anxiety scale, and stress scale at 0.91, 0.84, and at 0.90, respectively.

Students also answered the following questions related to some lifestyle habits:

On how many social networks do you have active accounts/profiles? (Facebook, Instagram, Twitter, Pinterest, Snapchat, WhatsApp, Tumblr, YouTube)? How many different devices do you use to access these profiles (a home computer, laptop, cell phone)? How many hours do you spend on social networks on average per day? How much sleep do you get on average at night (number of hours)? How often do you consume alcoholic beverages (Likert scale from 1–6, where 1 = never, 2 = very rarely, 3 = rarely, 4 = occasionally, 5 = frequently, and 6 = very frequently)? Is it less, the same or more compared to the period before the COVID-19 pandemic? How often do you consume psychoactive substances (on Likert scale from 1–6)? Is it less, the same or more compared to the period before the COVID-19 pandemic? Were you under home isolation during the COVID-19 pandemic due to infection or contact with an infected person (Yes or No)? To what extent did social networks provide you with an adequate replacement for the content that was denied to you during the pandemic (impossibility of gathering in catering facilities, cancellation of sports, cultural and other events, etc. – on a scale from 1 to 10)? The used surveys are provided in the Appendix.

### 2.4 Statistical analysis

We performed descriptive and univariate inferential statistical analysis to inspect the relationship between social media usage and studied mental health symptoms (namely, scores of depression and anxiety symptoms) in the context of the COVID19-pandemic. The statistical evaluation of the obtained results was accomplished by using the statistical software IBM SPSS Statistics (version 18.0). The research results were presented as tables and graphs.

The statistical analysis of the data included the application of descriptive statistics methods along with inferential statistics techniques (namely, Wilcoxon Signed Ranks test, Paired samples Student’s t-test and correlation tests). The descriptive statistics was applied to summarize the obtained findings. The results regarding numerical variables, such as lifestyle habits of the university students, were summarized as mean and standard deviations. The categorical variables were presented as frequency and percentages. Pearson correlation and Spearman’s rank correlations were applied to assess the level and significance of the association between the examined variables. Wilcoxon signed ranks test and Paired samples t-test were used to test the difference between the Phase 1 (initial phase) and the Phase 2 (follow up) distributions of the investigated variables, which were related to mental health issues. In all inferential statistics tests, the alpha level *p* < 0.05 was considered as the cut-off threshold for statistical significance.

### 2.5 Ethics

The research was conducted in concordance with the Declaration of Helsinki. All participants were informed about the study and they provided informed consent. This study research was approved by the Ethical Committee of the Faculty of Medicine of the University of Niš (no. 12-6,647-2/6).

## 3 Results

The Phase 1 survey was based on a sample of 1,476 participants, of whom were 714 males (48.4%) and 762 were females (51.6%) (2019/20). The Phase 2 (follow up point) (2021/22) survey included 1,400 participants, 692 female (49.4%) and 708 male students (50.6%).

**Graph 1:**
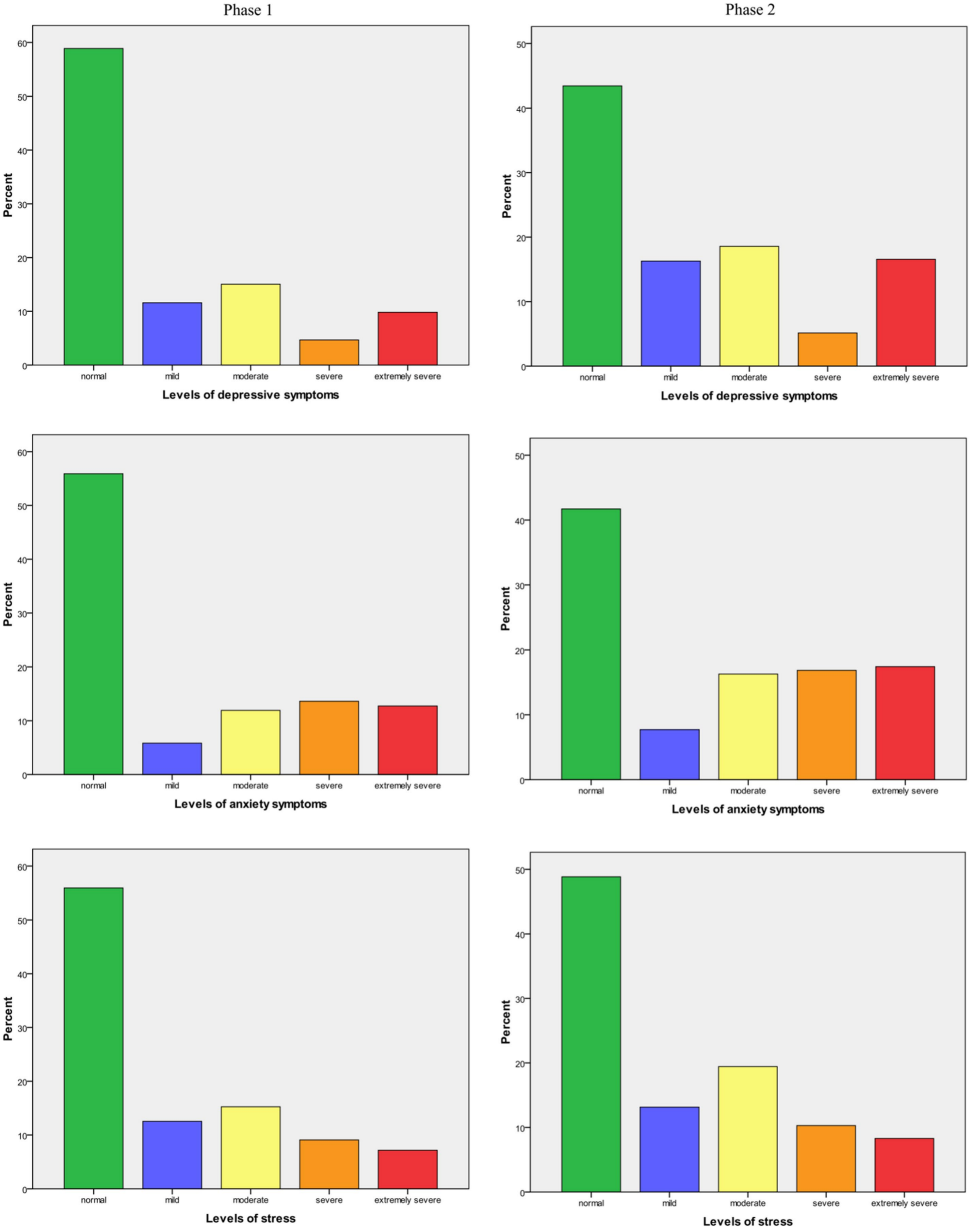
Scores of depressive and anxiety symptoms severity and stress levels at the phase 1 and at the phase 2 of the surveyed students.

Extremely severe levels of depression symptoms were observed for 145 students (9.9%), while both severe and extremely severe anxiety symptoms were observed for 389 students (26.4%), and severe and extremely severe levels of stress for 240 of them (16.2%) at the Phase 1 (Table 1; Graph 1).

**Table 1:** Scores of depressive and anxiety symptoms severity and stress levels (DASS 42) of the surveyed students at the Phase 1 (initial phase) and the Phase 2 (follow up after 2 years).

	Depression			Anxiety			Stress		
	Total	Female	Male	Total	Female	Male	Total	Female	Male
Symptoms at phase 1									
Normal	869	409	460	825	358	467	826	374	452
	58.9%	27.7%	31.2%	55.9%	24.3%	31.6%	55.9%	25.3%	30.6%
Mild	171	71	100	86	56	30	185	78	107
	11.6%	4.8%	6.8%	5.8%	3.8%	2.0%	12.5%	5.3%	7.2%
Moderate	222	141	81	176	89	87	225	165	60
	14.9%	9.4%	5.5%	11.9%	6.0%	5.9%	15.4%	11.3%	4.1%
Severe	69	53	16	201	134	67	134	75	59
	4.7%	3.6%	1.1%	13.6%	9.1%	4.5%	9.1%	5.1%	4.0%
Extremely severe	145	88	57	188	125	63	106	70	36
	9.9%	6.0%	3.9%	12.8%	8.5%	4.3%	7.1%	4.7%	2.4%
Symptoms at phase 2									
Normal	608	352	256	584	344	240	684	364	320
	43.4%	25.1%	18.3%	41.7%	24.6%	17.1%	48.9%	26.0%	22.9%
Mild	228	84	144	108	60	48	184	72	112
	16.3%	6.0%	10.3%	7.7%	4.3%	3.4%	13.1%	5.1%	8.0%
Moderate	260	116	144	228	68	160	272	144	128
	18.6%	8.3%	10.3%	16.3%	4.9%	11.4%	19.4%	10.3%	9.1%
Severe	72	40	32	236	92	144	144	48	96
	5.2%	2.9%	2.3%	16.9%	6.6%	10.3%	10.3%	3.4%	6.9%
Extremely severe	232	100	132	244	128	116	116	64	52
	16.5%	7.1%	9.4%	17.4%	9.1%	8.3%	8.3%	4.6%	3.7%

Levels of depression symptoms: normal (0–9), mild (10–13), moderate (14–20), severe (21–27), extremely severe (28+); Levels of anxiety symptoms: normal (0–7), mild (8–9), moderate (10–14), severe (15–19), extremely severe (20+); Levels of stress: normal (0–14), mild (15–18), moderate (19–25), severe (26–33), extremely severe (34+).

Extremely severe levels of depression symptoms were observed for 232 students (16.5%), while severe and extremely severe anxiety and stress symptoms were observed for 480 students (34.3%), and for 260 students (18.6%), respectively, at the Phase 2 survey (after 2 years) (Table 1; Graph 1). It could be emphasized here a significantly increase of extremely severe symptoms of depression (from 3.9 to 9.4%), and anxiety (from 4.3 to 8.3%) in male students than in females between Phase 1 to Phase 2 (Table 1).

As it is shown in Table 2, the severity scores of almost all six components of online social media addiction (BSMAS) of the surveyed students at the phase 2 were changed in comparison with the scores at the phase 1.

**Table 2:** Scores of online social media addiction (BSMAS) of the surveyed students at the phase 1 and the phase 2.

	Ls1 *n* (%)	Ls2 *n* (%)	Ls3 *n* (%)	Ls4 *n* (%)	Ls5 *n* (%)
BSMAS at phase 1					
BSMAS 1 salience	413	461	343	220	39
	28.0%	31.2%	23.3%	14.9%	2.6%
BSMAS 2 tolerance	268	526	461	153	68
	18.2%	35.6%	31.2%	10.4%	4.6%
BSMAS 3 mood modification	443	434	413	127	59
	30.0%	29.4%	28.0%	8.6%	4.0%
BSMAS 4 relapse	387	460	345	225	59
	26.2%	31.2%	23.4%	15.2%	4.0%
BSMAS 5 withdrawal symptoms	545	415	198	253	65
	36.9%	28.1%	13.5%	17.1%	4.4%
BSMAS 6 conflict	803	361	173	118	21
	54.4%	24.5%	11.7%	8.0%	1.4%
BSMAS at phase 2					
BSMAS 1 salience	488	472	212	180	48
	34.9%	33.7%	15.1%	12.9%	3.4%
BSMAS 2 tolerance	392	452	368	136	52
	28.0%	32.3%	26.3%	9.7%	3.7%
BSMAS 3 mood modification	400	256	428	228	88
	28.6%	18.3%	30.5%	16.3%	6.3%
BSMAS 4 relapse	388	396	276	240	100
	27.7%	28.3%	19.8%	17.1%	7.1%
BSMAS 5 withdrawal symptoms	660	276	172	264	28
	47.1%	19.7%	12.3%	18.9%	2.0%
BSMAS 6 conflict	692	264	260	116	68
	49.4%	18.9%	18.5%	8.3%	4.9%

Salience BSMAS 1 – I spend a lot of time thinking about social networks, what I am missing when I do not use them, or plan how I will use them; tolerance BSMAS 2 – I feel an overwhelming need to use social networks more and more; mood modification BSMAS 3 – I use social networks to escape from personal problems or to repair a bad mood; relapse BSMAS 4 – When I try to cut down the time spent on social networks, I fail; withdrawal symptoms BSMAS 5 – I become restless/anxious or disturbed if I am denied or abruptly cut off from social networks; conflict BSMAS 6 – I happen to use social networks to the extent that it adversely affects my job/ schooling (poor performance, poor concentration) or relationships with people (lie or argue in defense regarding the amount of time spent on or the way social networks are used). Ls1 to Ls5 – a five-point Likert scale (1=very rarely or never, 2=rarely, 3=sometimes, 4=often, 5=very often), and also a five-point Likert from 1-strongly disagree to 5-strongly agree, concerning experiences during the past year related to Social Media use in terms of BSMAS.

It was found that for Conflict and Relapse components of BSMAS severe and extremely severe scores were drastically increased in phase 2 – from 139 (9.4%) to 184 (13.2%) students who reported it, and from 284 (19.2%) to 340 (24.2%) students, respectively, while severe and extremely severe scores of Mood Modification were the most increased component of BSMAS among all of them in phase 2 – from 186 (12.6%) to 316 (22.6%) students who reported it (Table 2).

Severe and extremely severe scores of Tolerance, Salience and Withdrawal Symptoms component were very slightly decreased in phase 2 – from 221 (15.0%) to 188 (13.4%) students who reported it, from 259 (17.5%) to 228 (16.3%) students who reported it, and from 318 (21.5%) to 292 (20.9%) students, respectively (Table 2).

The relationship between the investigated lifestyle habits of the respondents and their corresponding mental health symptoms were examined by the Pearson’s correlation coefficient or by Spearman’s rho rank correlation coefficient. The resulting correlations’ coefficients are presented in Table 3.

**Table 3:** Correlations of some important variables regarding BSMAS and DASS42 scores at the phase 2.

	BSMAS1	BSMAS2	BSMAS3	BSMAS4	BSMAS5	BSMAS6	Depression	Anxiety	Stress
Gender	0.056^*^	−0.111^**^	−0.077^**^	0.012	−0.049	−0.059^*^	0.096^**^	0.116^**^	0.045
*p*	0.036	0.000	0.004	0.665	0.064	0.026	0.000	0.000	0.090
Age	−0.033	−0.027	−0.015	0.012	0.059^*^	−0.047	0.043	0.040	0.025
*p*	0.215	0.307	0.581	0.645	0.028	0.080	0.104	0.134	0.343
A1	−0.014	0.092^**^	0.057^*^	0.042	0.174^**^	0.123^**^	−0.031	0.171^**^	0.055*
*p*	0.604	0.001	0.033	0.120	0.000	0.000	0.249	0.000	0.039
A2	0.046	0.087^**^	0.043	0.101^**^	0.015	0.038	0.046	0.077^**^	0.023
*p*	0.088	0.001	0.108	0.000	0.571	0.152	0.083	0.004	0.392
A3	0.168^**^	0.255^**^	0.162^**^	0.236^**^	0.262^**^	0.235^**^	−0.009	0.042	0.007
*p*	0.000	0.000	0.000	0.000	0.000	0.000	0.745	0.117	0.803
A4	0.017	0.079^**^	0.061^*^	0.003	−0.032	−0.018	−0.055^*^	0.098^**^	0.028
*p*	0.517	0.003	0.023	0.918	0.238	0.491	0.038	0.000	0.290
A5	−0.009	−0.003	0.021	−0.050	0.023	0.020	−0.001	0.135^**^	0.055*
*p*	0.725	0.920	0.441	0.062	0.389	0.456	0.957	0.000	0.041
A50.1	0.042	0.094^**^	−0.030	0.045	−0.072^**^	−0.005	0.158^**^	0.126^**^	0.161**
*p*	0.117	0.000	0.268	0.092	0.007	0.852	0.000	0.000	0.000
A6	−0.040	−0.041	−0.016	−0.062^*^	0.065^*^	−0.009	0.156^**^	0.089^**^	0.190**
*p*	0.132	0.128	0.546	0.021	0.014	0.733	0.000	0.001	0.000
A60.1	0.037	0.064^*^	0.131^**^	0.044	−0.010	0.058^*^	0.012	0.044	−0.068*
*p*	0.166	0.017	0.000	0.099	0.715	0.030	0.655	0.096	0.011
A7	−0.041	−0.034	−0.098^**^	−0.113^**^	−0.131^**^	−0.083^**^	0.040	0.031	0.056*
*p*	0.122	0.203	0.000	0.000	0.000	0.002	0.131	0.246	0.035
A8	0.132^**^	0.119^**^	0.193^**^	0.025	0.232^**^	0.115^**^	−0.032	0.154^**^	0.035
*p*	0.000	0.000	0.000	0.347	0.000	0.000	0.230	0.000	0.195
Depression	0.071**	0.168**	0.185**	0.160**	0.088**	0.246**	1	0.642**	0.855**
*p*	0.008	0.000	0.000	0.000	0.001	0.000		0.000	0.000
Anxiety	0.142**	0.198**	0.319**	0.141**	0.197**	0.220**	0.642**	1	0.774**
*p*	0.000	0.000	0.000	0.000	0.000	0.000	0.000		0.000
Stress	0.126**	0.169**	0.210**	0.085**	0.126**	0.268**	0.855**	0.774**	1
*p*	0.000	0.000	0.000	0.001	0.000	0.000	0.000	0.000	

**Correlation is significant at the 0.01 level; *Correlation is significant at the 0.05 level. A1 – Number of active accounts/profiles; A2 – Number of active devices; A3 – How many hours do you spend on social networks on average per day? A4 – How much sleep do you get on average at night (number of hours)? A5 – How often do you consume alcohol (Likert scale from 1−6, where 1= never, 2= very rarely, 3= rarely, 4= occasionally, 5= frequently, and 6= very frequently)? A5.1 – Is it less, the same or more compared to the period before the COVID−19 pandemic? A6 – How often do you consume psychoactive substances (on Likert scale from 1−6)? A6.1 – Is it less, the same or more compared to the period before the COVID−19 pandemic? A7 – Were you under home isolation during the COVID−19 pandemic due to infection or contact with an infected person (Yes or No)? A8 – To what extent did social networks provide you with an adequate substitution for the content that was denied for you during the pandemic (impossibility of gathering in catering facilities, cancellation of sports, cultural and other events…) (on a scale of 1 to 10)?

The results indicate that the symptoms of depression are more profound in students who often frequently consumed alcoholic beverages and psychoactive substances during the COVID-19 pandemic (*r* = 0.16, *p* < 0.01, for both). Notably, anxiety symptoms were slightly more noticeable in respondents who slept longer hours during the night (*r* = 0.10, *p* < 0.01), consumed more amount of alcoholic beverages, particularly during the pandemic (*r* = 0.14, *p* < 0.01), along with the students to whom social networks supplied an adequate substitute for content which was not accessible in the course of the pandemic (*r* = 0.15, *p* < 0.01) (Table 3). Additionally, observed stress levels had the strongest correlations with consuming alcoholic beverages during the pandemic (*r* = 0.16, *p* < 0.01). Interestingly, stress was the most associated with the use of psychoactive substances (*r* = 0.19), but this correlation was reported to be slightly weaker and negative than in the pre-pandemic period (*r* = −0.07, *p* < 0.05). Between male and female participants of the survey, significant correlations patterns were detected in the presence of depressive symptoms (rho = 0.10, *p* < 0.01), and anxiety symptoms (rho = 0.12, *p* < 0.01), while the age of the participants did not have any impact. It can be summarized that there were many significant differences regarding the investigated parameters between the two phases (baseline and follow up) (Table 3). Also, findings from the same analysis have showed that increased symptoms of depression, anxiety and stress were positively correlated with all 6 BSMAS components in phase 2 (*p* < 0.01) (Table 3).

Difference in the scores of all six components of online social media addiction (BSMAS) were observed between the two studied phases. The application of the Wilcoxon Signed Ranks test revealed statistically significant decreased levels for all BSMAS scores (*p* < 0.01), except for BSMAS 4 – Relapse (“When I try to cut down the time spent on social networks, I fail”) (*p* = 0.14). It should be mentioned the fact that these are shifts (differences) for the period immediately before the official declaration of the pandemic and at its peak (two years after) (Table 4).

**Table 4:** Wilcoxon Signed Ranks test-based comparison of BSMAS variables between the phase 1 and the phase 2.

Phase 1 vs phase 2	BSMAS 1	BSMAS 2	BSMAS 3	BSMAS 4	BSMAS 5	BSMAS 6
Z	−3.622	−4.497	−5.624	−1.466	−3.629	−5.316
*p*	0.000	0.000	0.000	0.143 (n.s.)	0.000	0.000
*r*	0.07	0.08	0.11	n.s.	0.07	0.10

BSMAS 1 – salience; BSMAS 2 – tolerance; BSMAS 3 – mood modification; BSMAS 4 – relapse; BSMAS 5 – withdrawal symptoms; BSMAS 6 – conflict.

The Paired Samples *T*-Test assessed the mutual relationship between the influence of the investigated parameters immediately before the start of the pandemic (corresponding to the Phase 1), as well as in at its peak (corresponding to the Phase 2). A statistically significant increase was detected in Number of active accounts, and Time spent on social networks. In contrast, the analysis showed a decrease in Number of active devices (eta2 = 0.10 and 0.08, moderate influence). Relatedly, an increased levels of depression and anxiety were found, but with a smaller impact (eta2 = 0.033 and 0.028) (Table 5).

**Table 5:** Phase 1 vs phase 2 comparison of demographic and mental health variables based on paired samples student’s *t*-test results.

Phase 1 and follow up Phase 2	A1-number of active accounts	A2-number of active devices	A3-how many hours on social networks	Scores of depression symptoms	Scores of anxiety symptoms	Scores of stress symptoms
M1 (SD1)	3.17 (1.55)	2.13 (1.02)	6.25 (1.26)	1.98 (1.36)	2.23 (1.54)	2.00 (1.32)
M2 (SD2)	3.95 (1.90)	1.78 (0.57)	8.22 (1.57)	2.35 (1.48)	2.61 (1.57)	2.16 (1.35)
T	−12.529	11.127	−15.52	−6.807	−6.361	−3.189
Eta square	0.10	0.08	0.15	0.033	0.028	0.007
95% CI	−0.91 to−0.66	0.28 to 0.40	−0.97 to−0.16	−0.48 to−0.26	−0.49 to−0.26	−0.26 to−0.06

M1-mean at phase 1, SD1-standard deviation at phase 1; M2-mean at phase 2, SD2-standard deviation at phase 2.

## 4 Discussion

This work contributes to the existing literature on the COVID19 pandemics and sheds new light on the related mental health aspects of university students, and also of young people in general. The comparison between the two phases of our research (basic one and the follow up) provide novel insights into levels of probable anxiety and depression symptoms in the course of the pandemic and in the context of excessive Internet usage by university students. The results of this study suggests that the levels of depressive and anxiety symptoms, as well of stress levels among university students in Serbia were significantly increased during the pandemic, which was somewhat to be expected. Nevertheless, the presented findings should be evaluated and viewed in a broader context and in the light of previous studies by taking into account the published literature (including original research articles and systematic reviews).

Various scholars worldwide simultaneously obtained similar findings in their countries regarding this very vulnerable population, while some of them also pointed out to some physical health issues (28–35). In Chinese population the prevalence of depression, anxiety, sleep disorders, and posttraumatic stress symptoms was shown to be moderately high during COVID-19 crisis (21, 36–39), as it was among citizens in South Korea, India, Italy, and Denmark, where the authors showed similar results in their articles (40–43). However, Bélanger et al. (44) in Canada pointed out completely opposite findings, in contrast to their Canadian colleagues who confirm similar observations as in the previously mentioned countries (45). Interestingly, when it comes to comparison between US and China, according to Wang et al. (46), Americans reported more stress and depressive symptoms, while Chinese reported higher acute-traumatic stress symptoms. In our study, all three symptoms of mental disorders increased during the pandemic.

Alzahrani et al. (47) reported some socio-economic factors in a detailed systematic review and meta-analysis – their results indicate that risk factors of mental health problems were found to be female sex, younger age group, single/divorced marital status, lower education, lower income, unemployment status, but also university student status. According to Porter et al. (48) females also were affected the most, and the pandemic associated stressors such as health risks or expenses, economic adversity, food insecurity, and educational or employment disruption were found as risk factors for the development of more serious levels of probable anxiety and depression. Level of importance of these features showed variation cross countries, but anyway, prior parent/ peer relationships were identified as protective factors, and in contrast, long-term health or emotional problems were found as risk factors (48). Our results indicate that the symptoms of depression are more profound in students who often frequently consumed alcoholic beverages and psychoactive substances during the COVID-19 pandemic and anxiety symptoms were slightly more noticeable in respondents who slept longer hours during the night, consumed more alcoholic beverages, particularly during the pandemic, along with the students to whom social networks supplied an adequate substitute for content which was not accessible in the course of the pandemic. Besides, a significant increase of extremely severe symptoms of depression and anxiety was especially reported in male students.

Racine et al. (49) conducted exhaustive meta-analysis, highlighting drastic clinical manifestations of depression and anxiety disorders in adolescents during this observed pandemic period, as Shah et al. (50) also reported similar results in their study, but focusing on the total population of Canada, Great Britain and Pakistan. Nevertheless, the study by Ochnik et al. should be definitely singled out as they investigated mental health of the university students, and its differences between nine countries during the pandemic (Colombia, Czech Republic, Germany, Israel, Poland, Russia, Slovenia, Turkey, and Ukraine). According to their findings, the highest depression and anxiety risk occurred in Turkey. Notably, the lowest depression occurred in the Czech Republic and the lowest anxiety occurred in Germany (51). Our results were moderate to high when compared to this research.

At the bottom line, the results of the present study indicate that probable depression and anxiety levels were significantly higher among university students during COVID-19 pandemic. However, we do not exclude the possible influence of other factors on student’s mental health during the COVID-19 pandemic. Social isolation did not help university students in fulfilling their natural needs. In addition, online learning was stressful since most students were studying in a completely different environment which was not prepared for education. Students have indicated difficulty in focusing on academic work because of different sources of distraction. Students were more prone to be interrupted by their family members and household chores at home (52, 53). Females were influenced more by the pandemic than males regarding the depressive and anxiety symptoms, and younger age groups were more affected than older age groups (32). Both male and female students of our survey showed significant correlations patterns when it comes to the presence of depressive and anxiety symptoms, while the age of the participants did not have any impact. In the end, it should also be noted that COVID-19 pandemic had also increased the level of fear and worry about student’s’ own health and the health of their loved ones.

Finally, it should be noted that according to the recent research in Serbia, 4.1% of the total population and 10.8% of school and university students reported significant symptoms of depression, and 21.9% of the total population showed some worrying levels of anxiety symptoms (54). In the doctoral dissertation that dealt with the mental health of students from 12 faculties of the University of Kragujevac (Serbia), data were obtained that the prevalence of more pronounced depressive symptoms in the examined student population was 23.6%, while the prevalence of slightly more pronounced anxiety symptoms in the examined students was 33.6% (55). The development of more serious depressive symptoms was most significantly correlated with female gender, age, poor financial condition, housing conditions, and dissatisfaction with study conditions, while the development of more serious anxiety symptoms were in correlation with female gender, parents’ expectations of achieving an academic success and environmental pressure (55).

Đikanović et al. (56) even went a step further, hence the results of their research on the mental health of young people in Serbia, conducted in 2021 were somewhat alarming – namely, more than a third of the respondents (34.2%) indicated that they needed the services of a psychologist or psychotherapist. In a student environment, these potential problems are taken lightly. Young people often underestimate the need for conversation and professional help, or avoid them because of stigma and try to cope with their difficulties on their own. Unfortunately, the initial stages of the disorder are often unrecognized, and even serious symptoms or obvious signs of psychological problems are attributed to a subjective and transitory phase of the student lifestyle by the environment (54–56).

In this extremely challenging period of life, young people go through various psychological, physical and social changes that need to be overcome and find their place in the world of adults. Self-confidence and self-esteem are a significant source of overcoming the life and development crises of this age (56). Belief in one’s own strengths and resources enables an individual to cope with life’s challenges, so self-satisfaction and self-confidence are extremely important for psychological well-being and optimal functioning. Considering that, social networks are actually platforms where users present themselves to the world, the way they do it and the reactions they cause will affect their self-esteem, and thus their mental health.

Due to the insufficiently developed mental health services and mental health-associated social stigma, many students do not receive the required psychological support, and those affected from mental conditions do not always get adequate diagnosis and enough treatment (32, 52–54). Relatedly, the UNICEF information source on “Mental Health and Well-being in Serbia” states that “Those who suffer from mental health issues feel stressed, anxious and unmotivated, which together leads to decreased performance at university and puts them at risk of dropping out or engaging in risky behaviors, including substance use and unprotected sex. If not detected at an early stage, minor psychological issues may develop to mental health conditions that affect all spheres of a person’s life” (54).

### 4.1 Limitations and future directions

The main limitation of this study is that the research was conducted only in one university in Serbia and that the survey was based on self-reported mental health symptoms severity. In another words, the mental-health related findings rely only on the reported symptoms (more specifically, levels of depression, anxiety and stress), measured by DASS instrument, but not on clinical diagnosis. Such self-assessment approach may not be completely accurate.

Future comprehensive studies, based on larger cohorts, are anticipated to yield in-depth insight into public health issues associated with excessive Internet use in specific disease contexts. Possible directions for future research include, but not limited to, designing and applying more precise instruments to investigate how the Internet addiction is related to depression, anxiety and stress, and what is the underlying mechanism. Further studies in this direction are largely expected to claim more fruitful results.

## 5 Conclusion

The comparison of the studied variables between two phases brought to light a statistically significant increase in the number of active accounts, and time spent on social networks. In contrast, a decrease was observed in number of active devices used by students. Furthermore, increased symptoms of depression, anxiety, and stress levels were found. The detected patterns can serve as departing points to formulate new, more refined hypotheses in future studies.

Such pattern highlights the dynamic nature of the studied features and draws attention to changing conditions (such as the pandemics context). Despite the growing efforts to elucidate the impact of social media addiction on mental health, the underlying mechanism is still not fully understood as many aspects remain elusive. More multi-faceted and interdisciplinary studies are required to disentangle the complex nature of excessive Internet use.

## Data availability statement

The raw data supporting the conclusions of this article will be made available by the authors, without undue reservation.

## Ethics statement

The studies involving humans were approved by The Ethical Committee of the Faculty of Medicine of the University of Niš. The studies were conducted in accordance with the local legislation and institutional requirements. The participants provided their written informed consent to participate in this study.

## Author contributions

TJ, JV, and AV: conceptualization. AV and KK: methodology, software, and supervision. TJ, RM, and AV: validation and project administration. AV, KK, and TJ: formal analysis. TJ, JV, RM, and AV: investigation, resources, and data curation. TJ, JV, KK, and AV: writing, original draft preparation. KK, JV, RM, and TJ: review and editing. All authors contributed to the article and approved the submitted version.
