# Supplementary material for: A longitudinal study of the association between depression, anxiety and stress symptoms of university students in Serbia with excessive social media use before and during COVID-19 pandemic
Source: Front Public Health. 2023 Dec 18;11:1140961. doi: 10.3389/fpubh.2023.1140961 (PMC10757958; doi:10.3389/fpubh.2023.1140961)
Supplement: Supplementary file 1 [file Data_Sheet_1.pdf]

Dear colleagues,

We ask for your attention! Your answers are very valuable.

By completing the questionnaire, you will help us learn more about certain understudied effects of the Internet (social networks) on some aspects of health.

This questionnaire is anonymous, and the results will be used exclusively for scientific research purposes.

Gender Female Male

Birth Year ____________

PART I

*As an answer to the following questions, you should **enter** the appropriate number or **choose** one of the offered options.

1. On how many social networks do you have active accounts/profiles?

2. How many different devices do you use to access these profiles?

3. How many hours do you spend on social networks on average per day?

4. How much sleep do you get on average at night (number of hours)?

5. How often do you consume alcoholic beverages?

Never Very rarely Rarely Occasionally Frequently Very frequently

6. Is it less, the same or more compared to the period before the COVID-19 pandemic?

7. How often do you consume psychoactive substances?

Never Very rarely Rarely Occasionally Frequently Very frequently

8. Is it less, the same or more compared to the period before the COVID-19 pandemic?

9. Were you under home isolation during the COVID-19 pandemic due to infection or contact with an infected person? No Yes

10. To what extent did social networks provide you with an adequate replacement for the content that was denied to you during the pandemic (impossibility of gathering in public places, cancellation of sports, cultural events, etc.) – on a scale from 1 to 10?

PART II

*The following questions should be answered by **circling** the *number above* one of the descriptions provided that relates to the statement that precedes them.

1. I spend a lot of time thinking about social networks, what I am missing when I do not use them, or plan how I will use them.

5<br>Strongly agree	4<br>Agree	3<br>Neutral	2<br>Disagree	1<br>Strongly disagree

2. I feel an overwhelming need to use social networks more and more.

5<br>Very often	4<br>Often	3<br>Sometimes	2<br>Rarely	1<br>Very rarely or never

3. I use social networks to escape from personal problems or to repair a bad mood.

5<br>Very often	4<br>Often	3<br>Sometimes	2<br>Rarely	1<br>Very rarely or never

4. When I try to cut down the time spent on social networks, I fail.

5<br>Strongly agree	4<br>Agree	3<br>Neutral	2<br>Disagree	1<br>Strongly disagree

5. I become restless/anxious or disturbed if I am denied or abruptly cut off from social networks.

5<br>Strongly agree	4<br>Agree	3<br>Neutral	2<br>Disagree	1<br>Strongly disagree

6. I happen to use social networks to the extent that it adversely affects my job/ schooling (poor performance, poor concentration) or relationships with people (lie or argue in defense regarding the amount of time spent on or the way social networks are used).

5<br>Very often	4<br>Often	3<br>Sometimes	2<br>Rarely	1<br>Very rarely or never

PART III

*Please read each statement and circle a number 0, 1, 2 or 3 which indicates how much the statement applied to you over the past week. There are no right or wrong answers. Do not spend too much time on any statement.

The rating scale is as follows:

0 Did not apply to me at all

1 Applied to me to some degree, or some of the time

2 Applied to me to a considerable degree, or a good part of time

3 Applied to me very much, or most of the time

**1.**	I found myself getting upset by quite trivial things	0	1	2	3
**2.**	I was aware of dryness of my mouth	0	1	2	3
**3.**	I couldn't seem to experience any positive feeling at all	0	1	2	3
**4.**	I experienced breathing difficulty (eg, excessively rapid breathing, breathlessness in the absence of physical exertion)	0	1	2	3
**5.**	I just couldn't seem to get going	0	1	2	3
**6.**	I tended to over-react to situations	0	1	2	3
**7.**	I had a feeling of shakiness (eg, legs going to give way)	0	1	2	3
**8.**	I found it difficult to relax	0	1	2	3
**9.**	I found myself in situations that made me so anxious I was most relieved when they ended	0	1	2	3
**10.**	I felt that I had nothing to look forward to	0	1	2	3
**11.**	I found myself getting upset rather easily	0	1	2	3
**12.**	I felt that I was using a lot of nervous energy	0	1	2	3
**13.**	I felt sad and depressed	0	1	2	3
**14.**	I found myself getting impatient when I was delayed in any way (eg, elevators, traffic lights, being kept waiting)	0	1	2	3
**15.**	I had a feeling of faintness	0	1	2	3
**16.**	I felt that I had lost interest in just about everything	0	1	2	3
**17.**	I felt I wasn't worth much as a person	0	1	2	3

**18.**	I felt that I was rather touchy	0	1	2	3
**19.**	I perspired noticeably (eg, hands sweaty) in the absence of high temperatures or physical exertion	0	1	2	3
**20.**	I felt scared without any good reason	0	1	2	3
**21.**	I felt that life wasn't worthwhile	0	1	2	3
**22.**	I found it hard to wind down	0	1	2	3
**23.**	I had difficulty in swallowing	0	1	2	3
**24.**	I couldn't seem to get any enjoyment out of the things I did	0	1	2	3
**25.**	I was aware of the action of my heart in the absence of physical exertion (eg, sense of heart rate increase, heart missing a beat)	0	1	2	3
**26.**	I felt down-hearted and blue	0	1	2	3
**27.**	I found that I was very irritable	0	1	2	3
**28.**	I felt I was close to panic	0	1	2	3
**29.**	I found it hard to calm down after something upset me	0	1	2	3
**30.**	I feared that I would be "thrown" by some trivial but unfamiliar task	0	1	2	3
**31.**	I was unable to become enthusiastic about anything	0	1	2	3
**32.**	I found it difficult to tolerate interruptions to what I was doing	0	1	2	3
**33.**	I was in a state of nervous tension	0	1	2	3
**34.**	I felt I was pretty worthless	0	1	2	3
**35.**	I was intolerant of anything that kept me from getting on with what I was doing	0	1	2	3
**36.**	I felt terrified	0	1	2	3
**37.**	I could see nothing in the future to be hopeful about	0	1	2	3
**38.**	I felt that life was meaningless	0	1	2	3
**39.**	I found myself getting agitated	0	1	2	3
**40.**	I was worried about situations in which I might panic and make a fool of myself	0	1	2	3
**41.**	I experienced trembling (eg, in the hands)	0	1	2	3
**42.**	I found it difficult to work up the initiative to do things	0	1	2	3
